# Autophagic and lysosomal defects in human tauopathies: analysis of post-mortem brain from patients with familial Alzheimer disease, corticobasal degeneration and progressive supranuclear palsy

**DOI:** 10.1186/s40478-016-0292-9

**Published:** 2016-03-02

**Authors:** Antonio Piras, Ludovic Collin, Fiona Grüninger, Caroline Graff, Annica Rönnbäck

**Affiliations:** Karolinska Institutet, Department NVS, Center for Alzheimer Research, Division for Neurogeriatrics, Karolinska Institutet, SE-141 57 Huddinge, Sweden; Roche Pharma Research and Early Development, NORD DTA, Roche Innovation Center Basel, Basel, Switzerland; Department Geriatric Medicine, Genet. Unit, Karolinska University Hospital, Stockholm, Sweden

**Keywords:** Autophagy, Alzheimer disease, Tauopathy, Dementia, Hyperphosphorylated tau

## Abstract

**Introduction:**

The accumulation of insoluble proteins within neurons and glia cells is a pathological hallmark of several neurodegenerative diseases. Abnormal aggregation of the microtubule-associated protein tau characterizes the neuropathology of tauopathies, such as Alzheimer disease (AD), corticobasal degeneration (CBD) and progressive supranuclear palsy (PSP). An impairment of the lysosomal degradation pathway called macroautophagy, hereafter referred to as autophagy, could contribute to the accumulation of aggregated proteins. The role of autophagy in neurodegeneration has been intensively studied in the context of AD but there are few studies in other tauopathies and it is not known if defects in autophagy is a general feature of tauopathies. In the present study, we analysed autophagic and lysosomal markers in human post-mortem brain samples from patients with early-onset familial AD (FAD) with the APP Swedish mutation (APPswe), CBD and PSP and control individuals.

**Results:**

FAD, CBD and PSP patients displayed an increase in LC3-positive vesicles in frontal cortex, indicating an accumulation of autophagic vesicles. Moreover, using double-immunohistochemistry and *in situ* proximity ligation assay, we observed colocalization of hyperphosphorylated tau with the autophagy marker LC3 in FAD, CBD and PSP patients but not in control individuals. Increased levels of the lysosomal marker LAMP1 was detected in FAD and CBD, and in addition Cathepsin D was diffusely spread in the cytoplasm in all tauopathies suggesting an impaired lysosomal integrity.

**Conclusion:**

Taken together, our results indicate an accumulation of autophagic and lysosomal markers in human brain tissue from patients with primary tauopathies (CBD and PSP) as well as FAD, suggesting a defect of the autophagosome-lysosome pathway that may contribute to the development of tau pathology.

**Electronic supplementary material:**

The online version of this article (doi:10.1186/s40478-016-0292-9) contains supplementary material, which is available to authorized users.

## Introduction

Abnormal intracellular aggregation and accumulation of the microtubule-associated protein tau is a common feature of many neurodegenerative disorders, including Alzheimer disease (AD), frontotemporal lobar degeneration, Pick´s disease, argyrophilic grain disease, corticobasal degeneration (CBD) and progressive supranuclear palsy (PSP) [3]. Collectively, these neurodegenerative disorders are referred to as tauopathies. Under normal conditions, tau is predominantly expressed in neuronal axons where its main function is to promote microtubule assembly and stabilization, which is important for maintaining axonal transport and neuronal integrity [45, 60, 69]. The molecular mechanisms leading to intracellular tau aggregation in AD and other tauopathies are not fully understood, but abnormal post-translational modifications, such as hyperphosphorylation and acetylation [20, 47], and impaired degradation of tau [14] have been suggested.

Two major proteolytic systems contribute to tau degradation, the ubiquitin-proteasome system (UPS) and the autophagy-lysosome system [50, 52, 67]. The role of each of these pathways to the turnover of tau is an area of significant interest [16, 32, 38, 40, 66, 67]. The major lysosomal degradative pathway in eukaryotic cells, macroautophagy (hereafter referred to as autophagy), is responsible for degrading long-lived or aggregated proteins and is the principal mechanism for turning over cellular material too large to be degraded by the UPS [29, 75, 76]. Autophagy is a highly regulated process that involves the sequestration of cytoplasmic cargo, such as aggregated proteins and damaged organelles, within double-membrane vesicles called autophagosomes which are typically labelled by the microtubule-associated protein 1A/1B-light chain 3 (LC3) [23, 29, 30, 72, 76]. In order to achieve degradation of aggregated proteins in the autophagy-lysosome system, there must be a successful fusion of the autophagosomes with the lysosomes [17, 18, 29, 36, 73]. Functional autophagy is crucial for neuronal physiology and loss of autophagy in the central nervous system, for example by inactivation of key autophagy genes such as autophagy-related (*Atg*) proteins 5 (*Atg5*) or 7 (*Atg7*) leads to neurodegeneration [22, 31]. In *Atg7* knockout mouse brains, there is a significant accumulation of hyperphosphorylated tau suggesting a role of autophagy in the clearance of pathological tau in adult neurons [25]. Furthermore, increased accumulation of autophagic vesicles has been reported in human post-mortem AD brains and in mouse models of tauopathy [41, 54, 77]. Constitutive overexpression of mTor, (mammalian Target of rapamycin), a key negative regulator of the autophagic pathway, prevents activation of the autophagy pathway and increases the levels of hyperphosphorylated tau in a cell model of tauopathy [65]. Conversely, autophagy enhancers like rapamycin (an mTor inhibitor) or trehalose (an mTor-independent autophagy activator) can promote the degradation of insoluble tau in mouse models of tauopathy [9, 56, 61]. Finally, post-translational modifications of tau can interfere with and impair the clearance mechanisms. For example, phosphorylation of tau at serine 422 (Tau/pS422) prevents tau cleavage by caspase-3 at aspartic acid 421 (D421), precluding tau degradation by the autophagy-lysosome system [21]. Taken together these observations suggest that the autophagy-lysosome pathway plays an important role in the clearance of hyperphosphorylated tau. The majority of studies on human neurodegenerative disease and autophagy have included patients with Alzheimer disease where both Aβ and tau aggregations are key features (reviewed in [43, 55]), and only few studies have focused on other human tauopathies [19, 71]. Thus, in order to address the contribution of the autophagy-lysosomal system in different tauopathies, we studied human post-mortem brain tissue from patients with both tau and Aβ pathology [familial AD (FAD) cases with the Swedish double-mutation in the amyloid precursor protein (APPswe)] as well as brain tissue from patients with a primary tauopathies in the absence of significant amyloid pathology (CBD and PSP). In agreement with previous studies of sporadic AD cases [42, 51, 54], we found an accumulation of markers of the autophagy-lysosomal pathway in AD patients with the familial APPswe mutation. In addition, we showed that the autophagy-lysosomal system is impaired in patients with primary tauopathies suggesting that autophagic defects are a common feature of human tauopathies.

## Material and methods

### Brain samples

Human post-mortem brain tissue samples from frontal cortex were obtained from the Brain Bank at Karolinska Institutet. Three patients with early onset familial AD (FAD) caused by the Swedish amyloid precursor protein gene double-mutation KM670/671NL (APPswe), four patients with CBD and three patients with PSP as well as brain tissue from six control subjects (absence of neurodegenerative disease) were included (Table 1).

**Table 1:** Human brain samples

Case no	Age at death (years)	Gender (M/F)	Age at onset (years)	PMI (hours/days)	Brain weight (g)	Analysis
Ctrl_1	70	M	-	18 h	1482	IHC, IF
Ctrl_2	64	F	-	5 h	1204	IHC, IF, PLA, WB
Ctrl_3	84	F	-	96 h	1210	IHC, IF, PLA
Ctrl_4	59	M	-	na	1390	IHC, IF, PLA
Ctrl_5	82	F	-	9 h	1200	WB
Ctrl_6	80	M	-	16 h	1200	WB
APPswe_1	62	M	53	40 h	1024	IHC, IF, PLA, WB
APPswe_2	66	M	61	24 h	1140	IHC, IF, PLA, WB
APPswe_3	56	M	44	24 h	1161	IHC, IF, PLA, WB
CBD_1	80	F	74	8 days	1075	IHC, IF, PLA, WB
CBD_2	69	M	62	66 h	~1000	IHC, IF, PLA
CBD_3	70	M	64	24 h	930	IHC, IF, PLA
CBD_4	67	F	59	48 h	1045	WB
PSP_1	66	F	59	144 h	1490	IHC, IF, PLA
PSP_2	71	F	63	na	1121	IHC, IF, PLA
PSP_3	68	M	<66	na	1350	IHC, IF, PLA

*PMI* post-mortem interval, time from death until autopsy, *IHC* immunohistochemistry, *IF* immunofluorescence, *PLA* proximity ligation assay, *WB* western blot, *na* not available

### Immunohistochemistry

Immunohistochemical staining was performed on 5 μm sections from formalin fixed paraffin embedded (FFPE) frontal cortex of post-mortem brains. The sections were deparaffinised and hydrated through xylene and graded alcohol series. The sections were autoclaved with antigen retrieval buffer (DV2004, DIVA Decloaker, Biocare Medical) for 30 min at 110 °C (Decloaking Chamber NxGen, Biocare Medical). After the temperature decreased to room temperature (RT), sections were washed with water for 5 min and then in Tris-Buffered Saline (TBS) + 0.05 % Tween® 20 (TBS-T) (91414, Sigma-Aldrich). The sections were incubated in Peroxidase block solution (K4007, Dako) 5 min at RT to quench the endogenous peroxidase activity. Sections were then incubated with primary antibodies (Table 2) diluted in Antibody Diluent (S3022, Dako) for 45 min at RT, followed by 30 min incubation with EnVision Mouse (K4007, Dako) at RT. The immunoreactions were visualized with DAB (K4011, Dako). All sections were counterstained with haematoxylin for 30 s and washed in tap water. The sections were washed thoroughly in TBS-T between each step. Dehydration was performed in increasing concentrations of ethanol, cleared in xylene and mounted with DPX mountant (360294H, VWR). For every experiment, tissue sections from a control or a tauopathy-patient were incubated without the primary antibody to be used as a negative control.

**Table 2:** List of primary antibodies

Antigen	Supply	Reference	Clonality	Host	Dilution	Analysis
AT8	Thermo Scientific	MN1020	Monoclonal	Mouse	1:1000	IHC, PLA
Tau/pS422	Roche	-	Monoclonal	Mouse	1:1000	IF
LC3	Novus Biologicals	NB100-2331	Polyclonal	Rabbit	1:200	IF PLA
p62/SQSTM1	Santa Cruz	sc-25575	Polyclonal	Rabbit	1:50	IF
LAMP1	Santa Cruz	sc-20011	Monoclonal	Mouse	1:200	WB
Cathepsin D (Cat D)	Sigma-Aldrich	HPA003001	Polyclonal	Rabbit	1:500	IF
Cathepsin D (Cat D)	Abcam	Ab6313	Monoclonal	Mouse	1:500	IF
GAPDH	Enzo	ID4	Monoclonal	Mouse	1:2000	WB

*IHC* immunohistochemistry, *IF* immunofluorescence, *PLA* proximity ligation assay, *WB* western blot

For immunofluorescence staining, all sections were processed under the same standardized conditions, following the method described above with minor modifications. After deparaffinization and antigen retrieval, the sections were blocked with Background Punisher (BP974, Biocare Medical) for 10 min at RT followed by washing and incubation with primary antibodies (Table 2) in TBS-T (overnight 4 °C, humid chamber). After washing in TBS-T, sections were incubated (1 h at RT) with appropriate secondary antibodies [anti-mouse and anti-rabbit IgG (H + L)] conjugated to Alexa Fluor 546 (A-11003, Invitrogen) or Alexa Fluor 488 (A-11008, Invitrogen) at a concentration of 1:500 in TBS-T. After washing in TBS-T (3 × 10 min) slides were incubated with (or without) Sudan Black B (199664, Sigma-Aldrich), 5 min at RT to reduce autofluorescence. Sections were then washed in TBS-T and incubated with DAPI for 5 min (D9564, Sigma-Aldrich). After washing in TBS-T, coverslips were mounted with Vectashield Hard Set (H-1200, Vector Laboratories) and the slides were stored at 4 °C. Sections from controls and patients were also incubated without primary antibody and used as negative control. Sections were examined using a laser scanning confocal microscope (LSM 510 META, ZEISS), and images were acquired using the same settings (laser intensity, detector gain and amplifier offset).

### *In situ* Proximity Ligation Assay (PLA)

To specifically detect protein interaction (when the proteins are in close proximity <40 nm), PLA analyses were performed on section from FFPE frontal cortex of human brain from three patients of each group (Table 1). Deparaffinization and antigen retrieval were performed as described above. *In situ* PLA was performed according to the OLINK Bioscience instructions. To reduce non-specific signals, the sections were incubated with Blocking solution (82007, OLINK Bioscience) for 30 min at 37 °C. Sections were then incubated overnight at 4 °C with two primary antibodies (Table 2) in antibody diluent solution (82008, OLINK Bioscience) (80–100 μl/section). For each patient, two negative controls were performed by omitting one of the two primary antibodies (negative control 1 and negative control 2). After washing with TBS-T, sections were incubated with *in situ* PLA DNA-probes anti-rabbit *PLUS* and anti-mouse *MINUS* (82002 and 82004, OLINK, Bioscience) for 1 h at 37 °C. Ligation solution was added for 30 min at 37 °C, and washed twice with washing Buffer A (82047, OLINK, Bioscience). The amplification solution (92013, Detection reagents Far Red, OLINK Bioscience) was added for 100 min at 37 °C. Following incubation the sections were washed in Buffer B (82048, OLINK, Bioscience). The sections were allowed to dry and mounted with Vectashield Hard Set mounting medium with DAPI (H-1200, Vector Laboratories). Images from at least ten different fields of the frontal cortex for each sample were acquired using a laser scanning confocal microscope (LSM 510 Meta, ZEISS). Then, the images were analyzed with DUOLINK Image Tool software (OLINK Bioscience), which automatically counts the number of fluorescent signals (dots) per field. The signal was calculated as Dots = Dots_PLA_-(Dots_neg1_ + Dots_neg2_), where the Dots_PLA_ is the number of dots of *in situ* PLA and Dots_neg1_ and Dots_neg2_ are respectively the dots obtained from negative control 1 [only primary antibody 1, anti-tau clone AT8 (pS202/T205)] and negative control 2 (only primary antibody 2, LC3) [62]. Data were expressed as mean value ± Standard Deviation (S.D.) and ANOVA, followed by the Dunnett post-hoc test, was used for statistical analysis (IBM SPSS Software). Significance levels of *p < 0.05, **p < 0.01, *** *p* < 0.001 were used.

### Western blotting

Frozen tissue from frontal cortex (~145 mg/sample) of human brains from FAD (*n* = 3), CBD (*n* = 2) and Control (*n* = 3) (Table 1) were homogenized in RIPA buffer (50 mM Tris pH 7.5; 150 mM NaCl; 0.5 % sodium deoxycholate; 0.1 % SDS; 1 % NP40) containing Benzonase Nuclease (70664–3, Millipore, 1:1000), Phosphatase and Protease inhibitor cocktails (P8340 and P0044, Sigma-Aldrich, 1:100) on ice. Samples were centrifuged at 7000xg for 10 min at 4 °C. Supernatants were stored at −20 °C.

After determination of the protein content by BCA protein assay (23227, Thermo Fischer Scientific), homogenates (30–50 μg total protein) were separated on NuPAGE® 12 % Bis-Tris Gels, 1.0 mm, 10 wells (NP0341, Life Technologies) by electrophoreses in running buffer MOPS [3-(*N*-morpholino) propanelsulfonic acid] (NP0001, Life Technologies) and blotted onto iBlot Gel Transfer Stacks Nitrocellulose (IB301001, Life Technologies). The membranes were blocked in 5 % non-fat milk (70166, Sigma-Aldrich) in TBS-T. Membranes were incubated overnight at 4 °C with primary antibodies (see Table 2), washed in TBS-T and then incubated with secondary species-specific antibodies (NA934 anti-rabbit, NA931 anti-mouse, GE Healthcare) in TBS-T + 5 % non-fat milk for 1 h at RT. Blots were visualized with enhanced chemiluminescence reagent (34076, Pierce). Images were obtained with a FujiFilm LAS-3000 camera and semiquantitative analysis was performed using ImageJ software. Data were expressed as mean intensity of an arbitrary unit (AU) ± S.D. and were obtained from the average of at least 3 replications of the experiment. For quantification of immunoblots, protein levels were normalized to GAPDH. ANOVA, followed by the Dunnett post-hoc test, was used to compute statistical significance and significance levels of *p < 0.05, **p < 0.01, *** *p* < 0.001 were used.

## Results

### Characterization of tau pathology in frontal cortex from patients diagnosed with different tauopathies

**Fig. 1:**
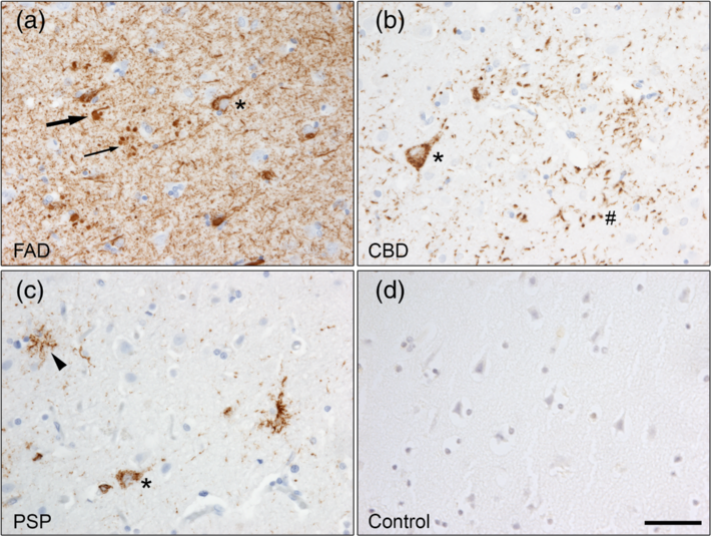
Tau pathology in frontal cortex as showed with the AT8 antibody. **a** APPswe mutation carriers show AT8 immunoreactivity in neurofibrillary tangles (arrow), pretangles (asterisk), neuropil threads and dystrophic neurites around amyloid plaques (small arrow). **b** AT8 immunohistochemistry revealed diffuse cytoplasmic tau immunoreactivity in neurons (pretangles, asterisk) and astrocytic plaques (#) in CBD patients, while **c** PSP patients show AT8 immunoreactivity in pretangles (asterisk) and tufted astrocytes (arrowhead). **d** No AT8 immunoreactivity was detected in control individuals. Scale bar: 50 μm

Human post-mortem brain tissue from cases with early-onset FAD, CBD, PSP and control individuals without neurodegeneration was analysed by immunohistochemistry to determine the presence of hyperphosphorylated tau using two different phospho-tau antibodies: clone AT8 (pS202/T205, Fig. 1) and Tau/pS422 (Additional file 1: Figure S1). Patients carrying the APPswe mutation showed extensive tau immunoreactivity in neurofibrillary tangles (Fig. 1a, large arrow), pretangles (defined by diffuse cytoplasmic tau immunoreactivity, Fig. 1a, asterisk), dystrophic neurites around amyloid plaques (Fig. 1a, small arrow) and neuropil threads. All CBD cases showed hyperphosphorylated tau in neuronal pretangles (Fig. 1b, asterisk), astrocytic plaques (Fig. 1b, #), threads in grey and white matter and oligodendroglial coiled bodies (data not shown). Tau-immunoreactive pretangle neurons (Fig. 1c, asterisk), tufted astrocytes (Fig. 1c, arrowhead) and coiled bodies in oligodendroglia (data not shown) were present in frontal cortex of the PSP cases. In contrast, no tau immunoreactivity was detected in the control individuals (Fig. 1d). Our data confirmed that hyperphosphorylated tau accumulates in the frontal cortex of all three patient groups.

### Accumulation of p62/SQSTM1 and colocalization with hyperphosphorylated tau in frontal cortex from cases with tauopathies

**Fig. 2:**
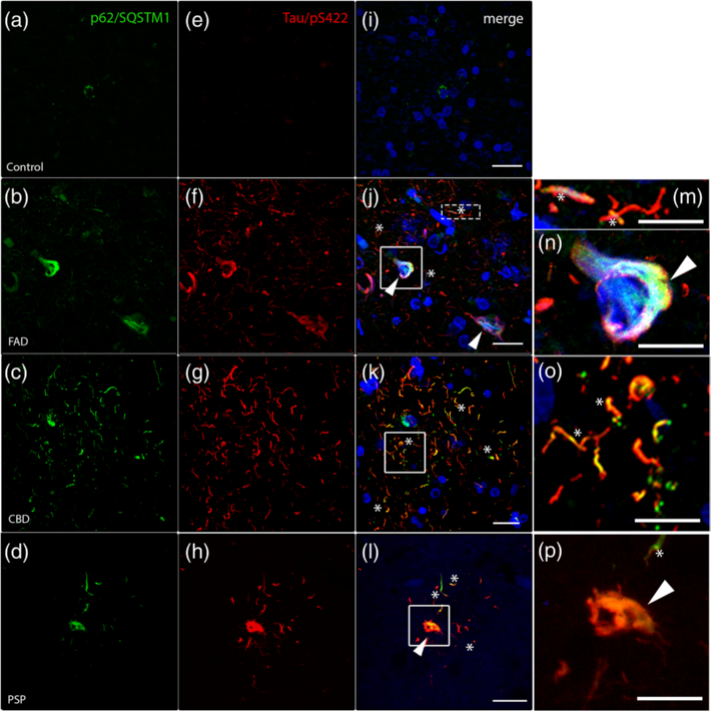
The autophagic marker p62/SQSTM1 accumulates and colocalizes with hyperphosphorylated tau in human tauopathies. Double immunohistochemistry against p62/SQSTM1 (green), hyperphosphorylated tau (Tau/pS422, red) and DAPI (blue) are shown by confocal analysis. **a** In control individuals, p62/SQSTM1-positive inclusions are only rarely observed and (**e**, **i**) no Tau/pS422 immunoreactivity were detected. **b**-**h** Strong immunoreactivity of p62/SQSTM1 (**b**-**d**) and Tau/pS422 (**f**-**h**) in post-mortem brain sections from FAD, CBD and PSP patients. **j**-**l** Merged pictures show colocalization between p62/SQSTM1 and Tau/pS422. **j** In FAD patients, colocalization is shown in neuronal threads (asterisks and high magnification **m**) and close to the nucleus (arrowheads and high magnification **n**). **k**, **o** In CBD patients, asterisks indicate colocalization in threads. **l**, **p** In PSP brains, colocalization is detected in threads (asterisks) and in some cell bodies (arrowheads). Scale: 20 μm (10 μm insert)

The polyubiquitin-binding protein p62/SQSTM1 is necessary to target protein aggregates for degradation via autophagy [6, 57], and/or the ubiquitin-proteasome system [2, 4, 63]. To investigate whether the protein clearance was defective in patients with tauopathies, we analysed the p62/SQSTM1 protein by immunostaining. Only rare p62/SQSTM1 inclusions were detected in control brain sections (Fig. 2a, green), while we observed an intense p62/SQSTM1 immunostaining in FAD, CBD and PSP (Fig. 2b-d, green), showing that p62/SQSTM1 accumulates in patients with tauopathies. Binding of p62/SQSTM1 to hyperphosphorylated tau is necessary for the clearance of tau as reported in p62-knockout mice that showed an accumulation of hyperphosphorylated tau and neurofibrillary tangles [2]. Therefore we investigated the colocalization of p62/SQSTM1 and phospho-tau (Tau/pS422, Fig. 2f-h). We found that p62/SQSTM1 colocalized with hyperphosphorylated tau in neuronal cell bodies (arrowheads in Fig. 2j, n) and in neuropil threads (asterisks in Fig. 2j, m) in FAD patients. In CBD patients, an extensive colocalization of p62/SQSTM1 and hyperphosphorylated tau was found mainly in threads (asterisks in Fig. 2k and high magnification Fig. 2o). Finally, in PSP patients we found colocalization of p62/SQSTM1 and hyperphosphorylated tau both in threads (asterisks in Fig. 2l, p) and in cell bodies (arrowhead in Fig. 2l, p), similar to what we observed in FAD patients. Altogether, the accumulation of p62/SQSTM1 and its colocalization with hyperphorylated tau suggest defective protein clearance in tauopathy patients, which is in accordance with previous studies [33, 34].

### Accumulation of LC3-positive structures in frontal cortex from patients with taoupathies and colocalization with hyperphosphorylated tau

**Fig. 3:**
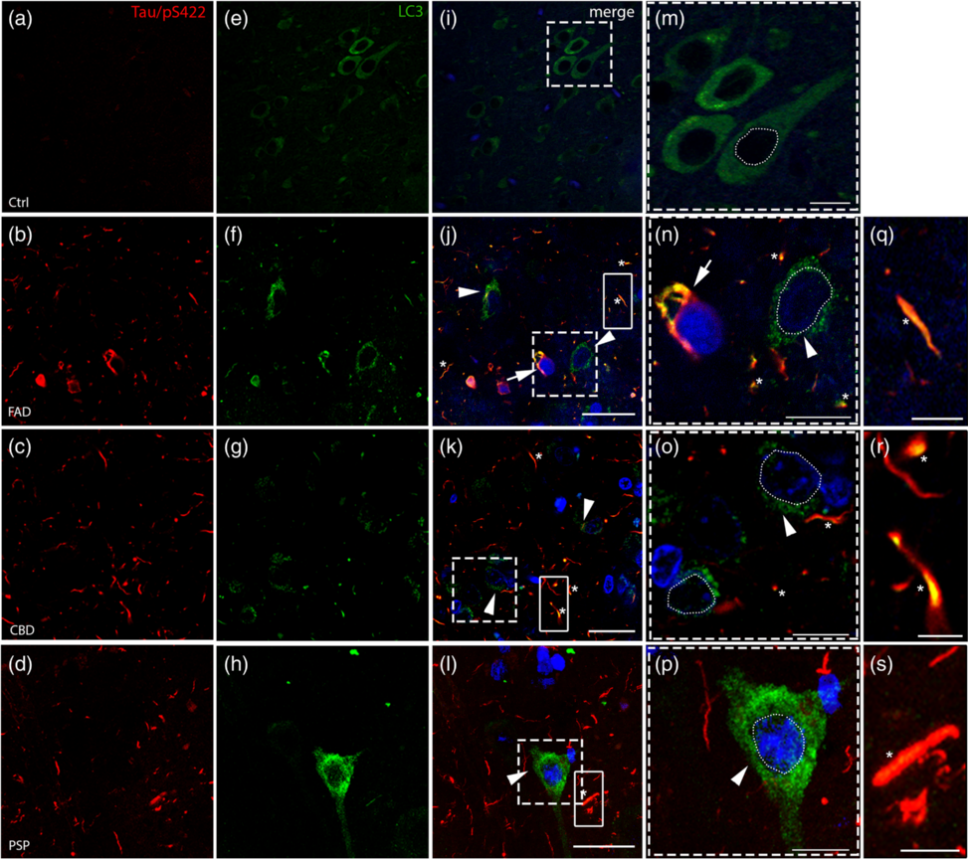
Accumulation of autophagic marker (LC3) and colocalization with hyperphosphorylated tau (Tau/pS422). Double immunofluorescence analysis on post-mortem tissues against (**a**-**d**) Tau/pS422 (red) and (**e**-**h**) LC3 (green). **j**-**p** Nuclei are stained with DAPI (blue, outlined with a white dashed line) in the merged pictures. **e** In control samples, LC3 staining is diffuse in the cytoplasm and LC3-positive dots are rarely observed (high magnification in (**m**) of the boxed area in **i**). **j**-**l** Arrowheads (and high magnifications, **n**-**p**) indicate cells showing accumulation of LC3 positive puncta in the perinuclear cytoplasm in FAD, CBD and PSP patients. Asterisks show colocalization in threads. In addition, FAD shows colocalization in tangle-like structures (**j**, **n**, arrows, see also SF. 2). **q**-**s** High magnification of the boxed areas in **j**-**l** showing colocalization of Tau/pS422 and LC3 in threads, respectively in FAD, CBD and PSP. Scale bars indicate 50 μm (**i**) and 25 μm (**j**-**l**); high magnification 10 μm (**m**-**p**) and 5 μm (**q**-**s**)

Several studies have shown that hyperphoshorylated tau can be cleared by the autophagy pathway [14, 16, 24, 66] and accumulation of pathological hyperphosphorylated tau and p62/SQSTM1 could result from defective autophagy-lysosomal clearance [35, 68]. To investigate whether the autophagy system is impaired in human tauopathies, we analysed the expression of LC3, a well-established autophagosome marker [29], by immunostaining of frontal cortex sections from controls and patients. In control individuals, we observed diffuse LC3 immunostaining in the cytoplasm and only rarely detected LC3-positive puncta (Fig. 3e, i, m). In contrast, in FAD patients (Fig. 3f, j, n), we observed an increase in LC3-positive puncta in the perinuclear cytoplasm (arrowheads in Fig. 3j, n) and in threads (asterisks in Fig. 3j). Interestingly, we also observed a marked increase of LC3-positive puncta in the cytoplasm of cells in the frontal cortex of CBD (Fig. 3g, k, o, arrowheads) and PSP patients (Fig. 3h, l, p, arrowheads). Altogether, these results suggest that accumulation of autophagic vesicles is a common feature in patients with tauopathies. In addition to the LC3-positive puncta in the perinuclear cytoplasm, we observed strong LC3 immunoreactivity in places distant from any clearly visible soma in all three patient groups but not in controls (Fig. 3).

Next we investigated if hyperphosphorylated tau colocalized with LC3 in patients with tauopathies. In FAD patients, colocalization between hyperphosphorylated tau (Tau/pS422) and LC3 was found in tangle-like structures (arrows in Fig. 3j, n; see also Additional file 2: Figure S2) and in neuropil threads (asterisks in Fig. 3j and high magnification Fig. 3n, q). CBD and PSP patients showed colocalization of hyperphosphorylated tau and LC3 mainly in threads (asterisks in Fig. 3k, l and high magnifications in Fig. 3o, r, s), although we cannot exclude that this is a result of the low levels of hyperphosphorylated tau in neuronal soma in CBD and PSP patients.

**Fig. 4:**
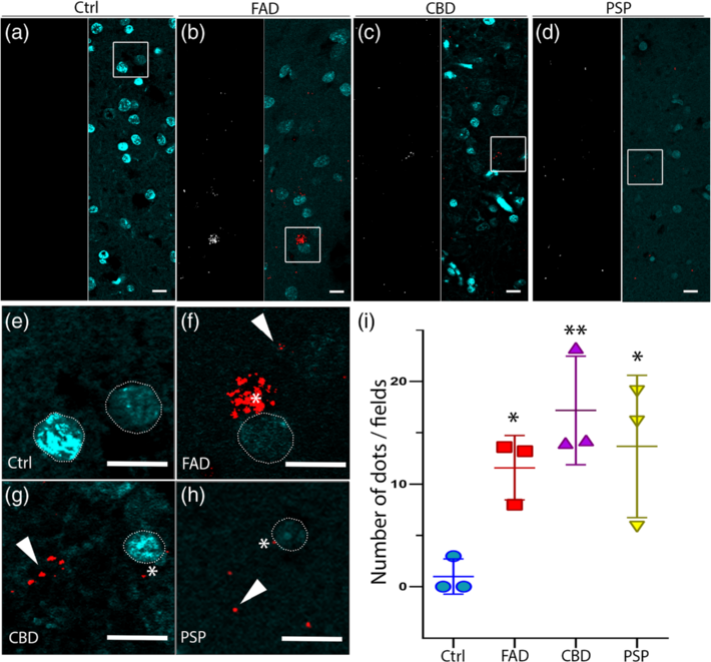
*In situ* PLA on human brain tissue confirms colocalization between hyperphosphorylated tau (AT8) and LC3. **a**-**d** The white signal shows the PLA dots in healthy controls and patients, respectively. In merged pictures, PLA staining (red) and DAPI (blue) are shown in FAD, CBD and PSP. PLA dots are rarely observed in control individuals. **e**-**h** High magnification of the boxed area from the merged pictures. Asterisks show PLA signal close to the nucleus (white dashed line), and arrowheads indicate PLA signal distant from clearly visible cell soma in FAD, CBD and PSP patients. Scale: 10 μm. **i** In the graphs PLA signal (number of dots/field) are indicated as Mean value ± S.D. from healthy controls and patients

To confirm the colocalization between hyperphosphorylated tau and LC3, we performed *in situ* proximity ligation assay (PLA) analysis, a technique to detect and quantify protein-protein interaction with high specificity and sensitivity [64]. We detected significant PLA signals for hyperphosphorylated tau (AT8) and LC3 in the frontal cortex of FAD, CBD and PSP cases (Fig. 4b-d), while no colocalization signal was observed in control individuals (Fig. 4a). The PLA signal was often observed in cell bodies (Fig. 4f, asterisk) and in the neuropil threads (arrowhead) in FAD patients. In contrast, CBD and PSP patients rarely showed PLA signal in the perinuclear region (Fig. 4g, h, asterisks), and mainly further away from cell nuclei (Fig. 4g, h, arrowheads), consistent with the double-immunofluorescence data. Quantification of the PLA signal (Fig. 4i) showed significantly more colocalization of hyperphosphorylated tau and LC3 in FAD (11.5 ± 2.2 dots/field), CBD (17.2 ± 5.3 dots/field) and PSP (13.9 ± 6.9 dots/field) compared to control individuals (0.7 ± 1.2 dots/field; *p* < 0.05 vs FAD, *p* < 0.01 vs CBD and *p* < 0.05 vs PSP). Altogether, our data suggest that pathological tau is present in LC3-positive structures in FAD, CBD and PSP human post-mortem brain.

### Accumulation of lysosomal markers and impairment of lysosomal integrity in tauopathies

**Fig. 5:**
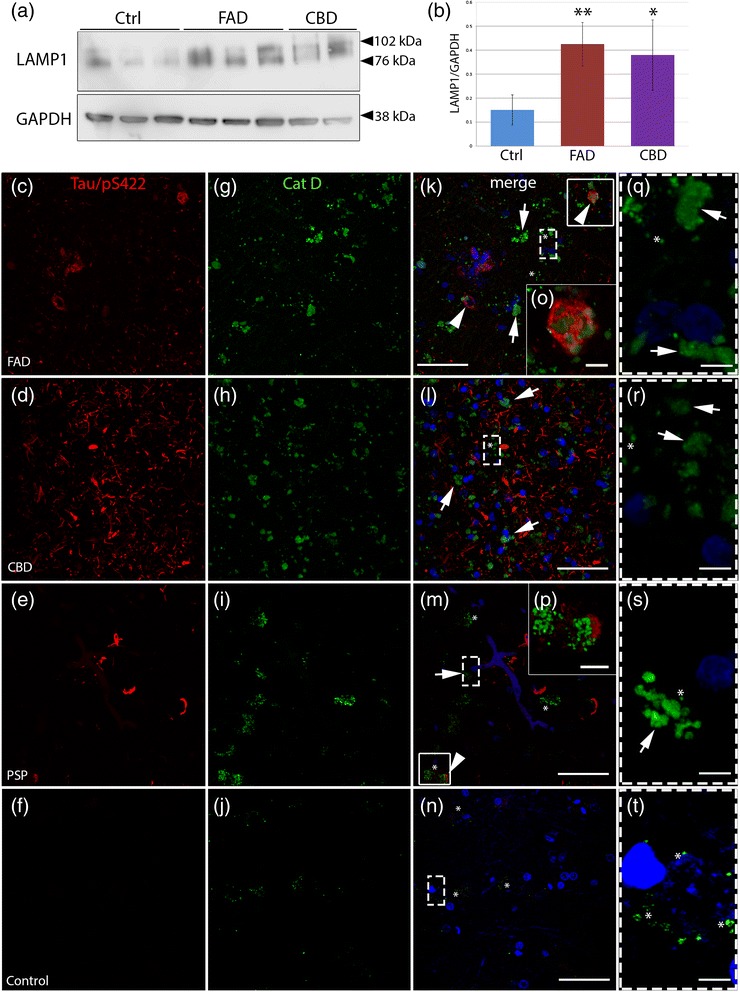
Accumulation of lysosomal markers and diffuse cytoplasmic Cat D immunoreactivity in tauopathies. **a** Western blot analysis of LAMP1 in human post-mortem brain (frontal cortex) extracts from control individuals (Ctrl, *n* = 3), early onset familial AD (FAD, *n* = 3) and CBD (*n* = 2). **b** LAMP1 levels quantified by densitometry and normalized to GAPDH in Ctrl, FAD and CBD. Results are shown as the mean value of arbitrary unit ± S.D (mean of three independent experiments). **c**-**t** Double immunohistochemistry against hyperphosphorylated tau (Tau/pS422, red, **c-f**) and Cat D (HPA003001, Sigma-Aldrich) (green, **g**-**j**) are shown by confocal analysis. **k**-**n** Merged pictures with nuclear staining (DAPI, blue). In FAD, CBD and PSP cases, strong Cat D-immunoreactivity is present in the soma of the cells in frontal cortex compared to the control. **k**-**m** (and high magnification **q**-**s**) Distinct vesicle-like structures (asterisks) and diffuse immunoreactivity (arrows) of Cat D throughout the cell bodies are shown in the pictures. In addition, in FAD and PSP, arrowheads in **k** and **m** and high magnification pictures (**o**-**p**) of boxed areas show hyperphosphorylated tau-positive structures close to Cat D-positive structures. **t** Brain tissues from control individuals show few Cat D-positive vesicle structures (asterisks). Scale bars: 50 μm (5 μm high magnification)

The accumulation of autophagic vesicles in AD patients has been suggested to be a result of defective lysosomal clearance [37, 39, 70]. To investigate this hypothesis, we analysed the localisation and the integrity of lysosomes in frontal cortex of tauopathy patients. First, we analysed the protein level of the lysosomal-associated membrane protein 1 (LAMP1), a major lysosomal glycoprotein, by Western blot. We found significantly higher LAMP1 protein levels in frontal cortex from FAD (AU 0.42 ± 0.09) and CBD (AU 0.38 ± 0.03) compared to control (AU 0.15 ± 0.062, *p* < 0.01 vs FAD and *p* < 0.05 vs CBD, Fig. 5a-b). Frozen brain tissue for Western blot analysis was not available from patients with PSP.

Next, we analysed the expression of Cathepsin D (Cat D), a major lysosomal hydrolase involved in the proteolytic degradation of proteins in lysosomes, by immunofluorescence. In frontal cortex from controls, we observed small perinuclear Cat D-positive punctate structures, corresponding to lysosomes (Fig. 5j, n, t, asterisks and Additional file 3: Figure S3). In contrast, the Cat D immunoreactivity was stronger in FAD, CBD and PSP patients compared to control individuals (Fig. 5g-i and Additional file 3: Figure S3). Two different patterns of Cat D immunoreactivity were observed: (1) a staining pattern of small vesicular structures, corresponding to lysosomes (Fig. 5q-s, asterisks) and (2) a diffuse Cat D staining pattern showing a fluorescent signal throughout the cytoplasm (Fig. 5q-s, arrows, and Additional file 3: Figure S3), suggesting that Cat D could have leaked out of the lysosomes (reviewed in [8, 74, 78]). Taken together the Cat D results suggest that the lysosomal integrity may be impaired in all three patient groups.

Finally we analysed the colocalization of hyperphosphorylated tau with Cat D using double immunofluorescence. In all three patient groups, we found that Cat D-positive staining rarely colocalized with hyperphosphorylated tau and only a few cells were immunoreactive for both Tau/pS422 (red) and Cat D (green) (arrowheads and inset in FAD and PSP, Fig. 5k, m, o, p). This suggests that hyperphosphorylated tau is not localized to lysosomes which is in line with previous studies in sporadic AD patients [5, 15].

## Discussion

Here we used human post-mortem brain tissue to demonstrate that abnormal accumulation of markers of the autophagy-lysosomal pathway is a common feature of different tauopathies. We found that patients with primary (CBD and PSP) and secondary (early onset FAD) tauopathies show an abnormal accumulation of the autophagy markers p62/SQSTM1 and LC3, and that both these markers colocalized with hyperphosphorylated tau. In FAD patients, hyperphosphorylated tau colocalized with LC3-positive structures and p62/SQSTM1 both in the soma and in neuritic threads, while in CBD and PSP patients, colocalization was found mainly in threads. Previous studies have demonstrated that hyperphosphorylated tau impairs microtubule-based axonal retrograde transport [27, 44, 46, 49], and that disruption of microtubule-based vesicle transport, either by the microtubule-depolymerizing drug vinblastine or by deleting the motor-protein dynein, results in a massive accumulation of autophagosomes in neurites [7, 28]. Thus, it is tempting to speculate that the accumulation of hyperphosphorylated tau, p62/SQSTM1 and LC3 in threads in the tauopathy patients are, at least partly, due to impaired axonal transport. It should be noted that in CBD and PSP patients, hyperphosphorylated tau is found both in neurons and in glial cells, and although the autophagy-lysosomal pathway has been demonstrated to be the main degradative pathway of tau in neurons, other mechanisms (including the ubiquitin-proteasome system) may be involved in degradation of tau in other cell types such as glia [32, 67, 68].

Our analysis of lysosomal markers showed increased protein levels of LAMP1 and Cat D in FAD patients as well as in patients with primary tauopathies, suggesting a defective lysosomal clearance which is in line with previous reports in AD brain [10, 12, 48, 53, 58]*.* In contrast to the control cases where Cat D staining was typically punctate, indicating localization within lysosomes*,* we observed diffuse cytoplasmic Cat D immunoreactivity in the tauopathy patients. Diffuse Cat D staining in the soma is indicative of lysosomal membrane rupture [1], and can be induced by oxidative stress [26, 59]. It has also been reported that tau can interact with lysosomal membranes and trigger lysosomal permeability in vitro [68] and that small tau fibrils can bind to lysosomal membranes resulting in lysosomal damage in a transgenic mouse model of AD [15]. Further studies are needed to determine if there is a leakage of Cat D into the cytoplasm in human tauopathies, but our data indicate that defective lysosomal integrity is prominent in FAD, CBD and PSP patients. It should be noted that the increased LAMP1 levels could be attributable to gliosis in the tauopathy patients [5, 11, 13].

In recent years, several studies have indicated that (pharmacological) induction of autophagy can be beneficial for treatment of neurodegenerative diseases, and increased autophagy has been shown to ameliorate pathology in various disease models by enhancing the clearance of intracytoplasmic protein aggregates, including hyperphosphorylated tau [22, 66]. It is possible that an impaired retrograde transport could explain the observed accumulation of LC3-positive structures containing hyperphosphorylated tau in threads in tauopathy patients. On the other hand, our findings from the FAD cases suggest that hyperphosphorylated tau can be transported to the soma where we observed colocalization with LC3 and p62/SQSTM1.

## Conclusions

In conclusion, our findings give support for an impairment of the autophagy-lysosomal system in patients with primary tauopathies as well as in familial AD caused by the Swedish APP mutation. Although the autophagosomal-lysosomal clearance pathway is compromised in all three tauopathies, our observations also indicate that the precise location of the impairment along this pathway is not necessarily the same. Thus, our study highlights the fact that therapeutic strategies targeting the autophagosomal-lysosomal pathway may need to be specifically tailored for different tauopathies.

## Ethical approval

All procedures performed in studies involving human participants were in accordance with the ethical standards of the institutional and/or national research committee (the Regional Ethical Review Board in Stockholm, Sweden) and with the 1964 Helsinki declaration and its later amendments or comparable ethical standards.

## Additional files

Additional file 1: Figure S1. Tau pathology observed with the Tau/pS422 antibody. a-f Immunofluorescence staining against hyperphosphorylated tau (Tau/pS422) in human brain samples. Intense immunoreactivity in FAD, CBD and PSP. a FAD cases show immunoreactivity in neurofibrillary tangles, pretangles, neuropil threads and dystrophic neurites. b-c CBD and PSP cases show immunoreactivity in neurons and glial cells. d-f High magnification of the boxed areas in a-c. Scale bars: 50 μm (10 μm high magnification) (TIF 3632 kb) Additional file 2: Figure S2. Colocalization between hyperphosphorylated tau (Tau/pS422) and LC3 in AD cases. a-d Double immunofluorescence staining against hyperphosphorylated tau (a) and LC3 (b) and merge (c) pictures in human brain samples show colocalization in neurons (NFT-like structures) in FAD samples. d Superposition of confocal stacks. Scale bar: 10 μm (TIF 25517 kb) Additional file 3: Figure S3. Diffuse staining pattern of Cathepsin D (Cat D) (ab6313, Abcam) in tauopathies a-h Merged pictures show immunofluorescence staining of Cat D (green) and DAPI (nuclear marker, blue). a-c Diffuse immunoreactivity for Cat D in FAD, CBD and PSP patients (arrows) and distinct vesicle-like structures (asterisks). d and h Control samples show Cat D-positive vesicular-like structure (asterisks) and not diffuse staining. e-h High magnification of the boxed areas in a-d. Scale bars: 50 μm (20 μm high magnification). (TIF 25515 kb)
