# Supplementary figures and images for: Autophagic and lysosomal defects in human tauopathies: analysis of post-mortem brain from patients with familial Alzheimer disease, corticobasal degeneration and progressive supranuclear palsy
Source: Acta Neuropathol Commun. 2016 Mar 2;4:22. doi: 10.1186/s40478-016-0292-9 (PMC4774096; doi:10.1186/s40478-016-0292-9)

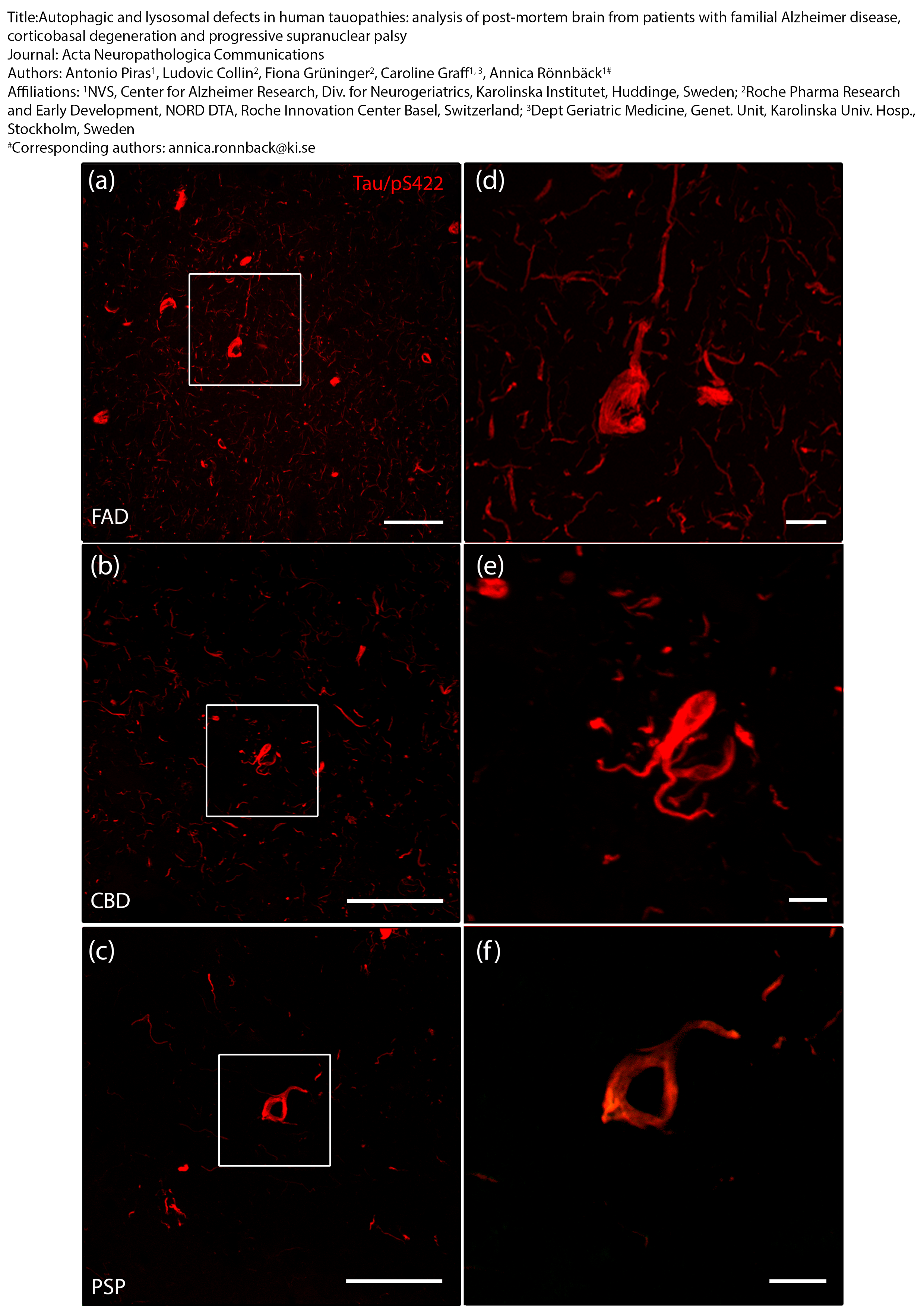

Supplement: Additional file 1: Figure S1. — Tau pathology observed with the Tau/pS422 antibody. a-f Immunofluorescence staining against hyperphosphorylated tau (Tau/pS422) in human brain samples. Intense immunoreactivity in FAD, CBD and PSP. a FAD cases show immunoreactivity in neurofibrillary tangles, pretangles, neuropil threads and dystrophic neurites. b-c CBD and PSP cases show immunoreactivity in neurons and glial cells. d-f High magnification of the boxed areas in a-c. Scale bars: 50 μm (10 μm high magnification) (TIF 3632 kb) [file 40478_2016_292_MOESM1_ESM.tif]

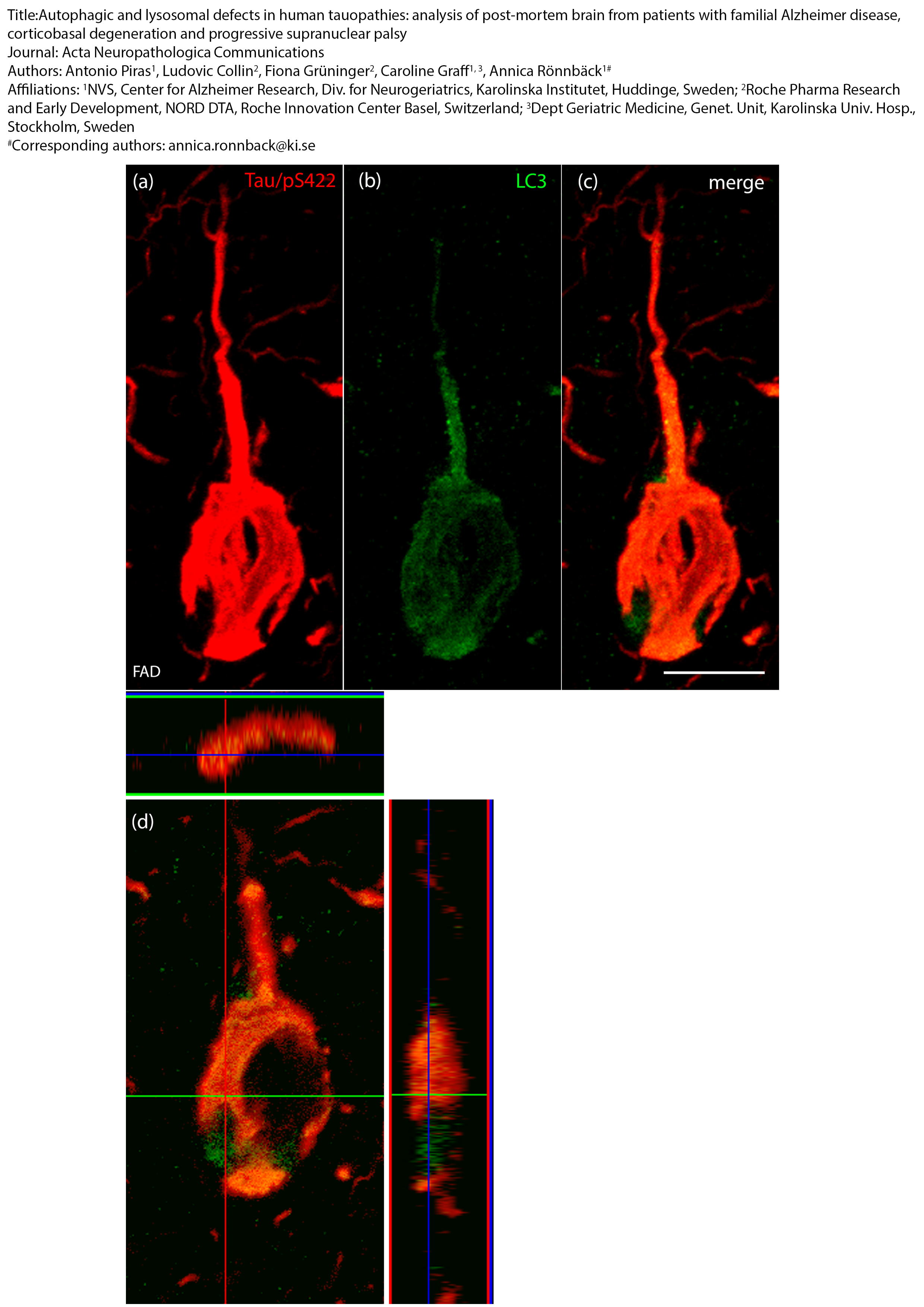

Supplement: Additional file 2: Figure S2. — Colocalization between hyperphosphorylated tau (Tau/pS422) and LC3 in AD cases. a-d Double immunofluorescence staining against hyperphosphorylated tau (a) and LC3 (b) and merge (c) pictures in human brain samples show colocalization in neurons (NFT-like structures) in FAD samples. d Superposition of confocal stacks. Scale bar: 10 μm (TIF 25517 kb) [file 40478_2016_292_MOESM2_ESM.tif]

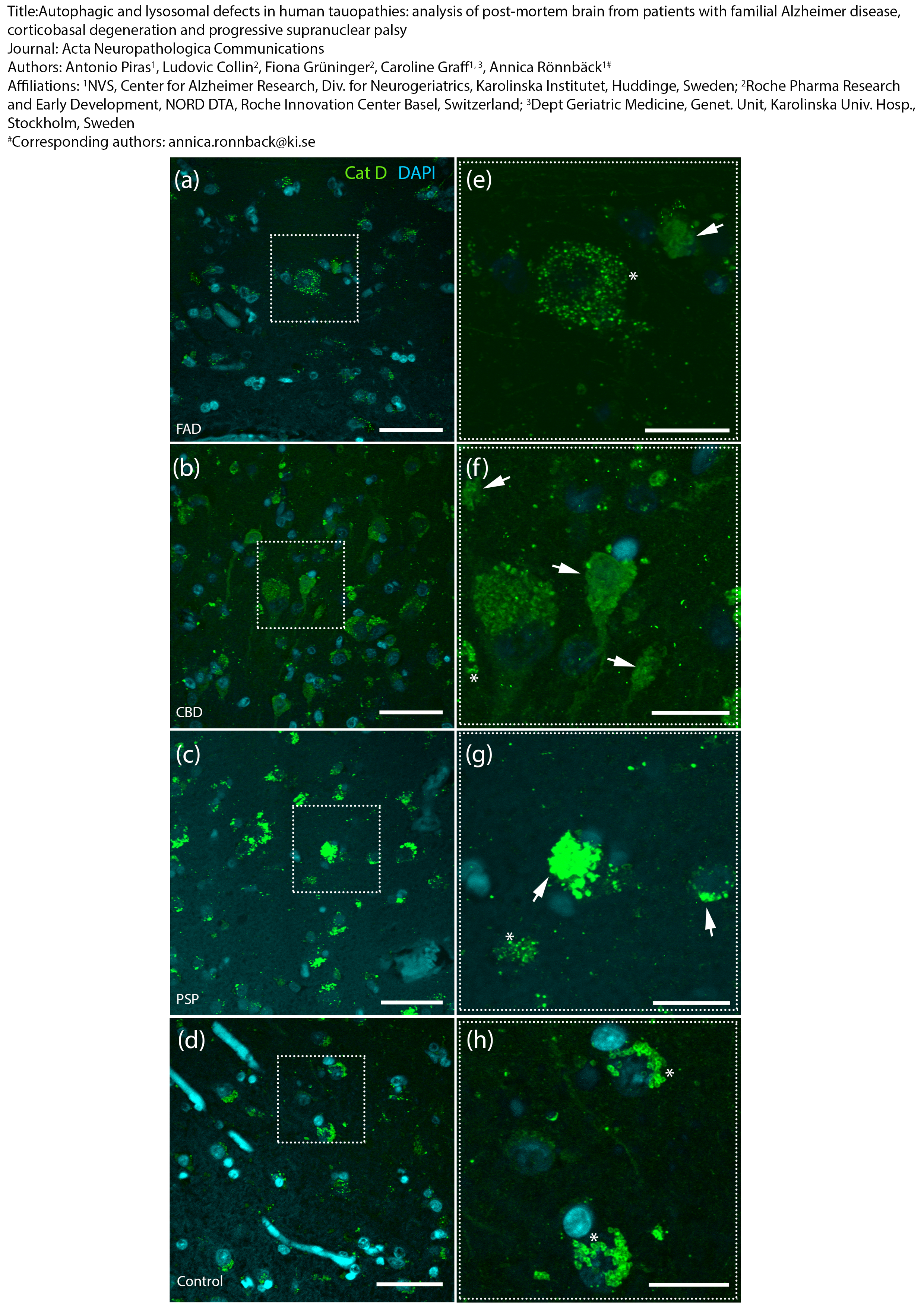

Supplement: Additional file 3: Figure S3. — Diffuse staining pattern of Cathepsin D (Cat D) (ab6313, Abcam) in tauopathies a-h Merged pictures show immunofluorescence staining of Cat D (green) and DAPI (nuclear marker, blue). a-c Diffuse immunoreactivity for Cat D in FAD, CBD and PSP patients (arrows) and distinct vesicle-like structures (asterisks). d and h Control samples show Cat D-positive vesicular-like structure (asterisks) and not diffuse staining. e-h High magnification of the boxed areas in a-d. Scale bars: 50 μm (20 μm high magnification). (TIF 25515 kb) [file 40478_2016_292_MOESM3_ESM.tif]
